# Enhanced Stability of Indocyanine Green by Encapsulation in Zein-Phosphatidylcholine Hybrid Nanoparticles for Use in the Phototherapy of Cancer

**DOI:** 10.3390/pharmaceutics13030305

**Published:** 2021-02-26

**Authors:** Eun-Hye Lee, Mi-Kyung Lee, Soo-Jeong Lim

**Affiliations:** 1 Department of Integrated Bioscience and Biotechnology, Sejong University, Seoul 05006, Korea; 2 Department of Pharmaceutical Sciences, Woosuk University, Jeonju 55338, Korea; leemk85@woosuk.ac.kr

**Keywords:** indocyanine green, zein, phosphatidylcholine, photodynamic therapy, hybrid nanoparticles

## Abstract

Indocyanine green (ICG) is a clinically approved near-infrared dye that has shown promise as a photosensitizer for the phototherapy of cancer. However, its chemical instability in an aqueous solution has limited its clinical application. Encapsulating ICG in liposomes, phosphatidylcholine nanoparticles (PC-NP), has shown partial effectiveness in stabilizing it. Prompted by our recent finding that the zein-phosphatidylcholine hybrid nanoparticles (Z/PC-NP) provide an advanced drug carrier compared to PC-NP, we herein investigated the potential of Z/PC-NP as an improved ICG formulation. Dynamic light scattering analysis, transmission electron microscopy, and Fourier-transform infrared spectroscopy studies showed that ICG was encapsulated in Z/PC-NP without hampering the high colloidal stability of the Z/PC-NP. During storage, the Z/PC-NP almost completely inhibited the ICG aggregation, whereas the PC-NP did so partially. The Z/PC-NP also more effectively blocked the ICG degradation compared to the PC-NP. The phototoxicity of ICG encapsulated in Z/PC-NP on cancer cells was twofold higher than that in the PC-NP. The ICG encapsulated in Z/PC-NP, but not in PC-NP, maintained its photocytotoxicity after four-day storage. These findings highlight the promising potential of Z/PC-NP as an ICG formulation that provides a higher stabilization effect than PC-NP.

## 1. Introduction

Indocyanine green (ICG) is a near-infrared (NIR) fluorescent dye that was approved by the FDA in 1959. It has been used for medical diagnoses to determine cardiac output, hepatic function, and gastric blood flow, and it has been used for intraoperative imaging and sentinel lymph node mapping [1,2]. The advantages of NIR light include its deep tissue penetration and the low autofluorescence of biological tissues [3,4], and the strong NIR absorbing property of ICG has recently led to broadening its potential applications to phototherapy; ICG taken up by tumor cells produces reactive oxygen species upon NIR light irradiation, leading to cell death [5]. Photodynamic/photothermal therapy using ICG as a photosensitizer is currently under intensive preclinical and clinical investigation as a promising cancer treatment modality with minimal systemic toxicity [6]. The potential application of ICG in tumor phototherapy, however, is limited by its physicochemical drawbacks. ICG is prone to aggregation and degradation in an aqueous solution [7], and such physicochemical transformations of the ICG lead to a shift in the maximum absorption wavelength, self-quenching, and decreased emission intensity [7,8]. To overcome these limitations, attempts have been made to encapsulate ICG in various nanocarriers, including micelles, polymeric nanoparticles, and liposomes [9,10]. Among these carriers, liposomes—namely, phosphatidylcholine nanoparticles (PC-NP)—are particularly promising drug carriers due to the biocompatibility of phospholipids [11,12]. Several attempts have been made by researchers to develop liposomal ICG for diagnostic imaging purposes (e.g., as a probe for lymph node imaging) [13,14], and recently for therapeutic purposes [10,15,16].

A high drug-loading capacity is a critical factor in the clinical development of nanoparticle formulations to be of an administrable and cost-effective dosage form [17,18]. Since ICG is a water-soluble amphiphilic dye composed of lipophilic polyaromatic polyene groups and hydrophilic sulfonate groups [19], ICG can be effectively encapsulated in liposomes either in the inner aqueous compartment and/or within the phospholipid membranes [20]. However, studies have demonstrated that the stability of liposomal ICG is very dependent on the ratio of ICG to lipids [20]. It appears that the ratio of ICG to lipids should be less than 0.4 mol% to retain ICG stability [15]. When the ICG to lipid ratio was 0.38 mol%, the liposomal encapsulation was sufficient to stabilize the ICG [13]. In contrast, when it was much higher (1.5~4.2 mol%), the ICG stabilization by liposomal encapsulation was limited [19,21]. In this regard, we recently reported that the surface coating of ICG-encapsulated liposomes with chitosan greatly improved the ICG stability despite the ratio of 4.2 mol% of ICG to lipids [19]. This seems likely to be mediated by electrostatic interaction between negatively charged ICG molecules and positively charged chitosan molecules. Chitosan coating, however, resulted in a substantial increase in the liposomal size, together with greatly increased heterogeneity, limiting its clinical development [19]. One of the strategies available may be the co-incorporation of cationic components together with ICG in the liposomal membranes to improve the ICG stabilization effect without affecting the particle size.

Zein is a corn protein with a “Generally Recognized As Safe” status approved by the FDA. Zein is amphiphilic but water-insoluble due to the strong hydrophobic properties caused by its high content of hydrophobic amino acids [22]. It is a mixture of α, β, and γ isoforms and shows unique structural transformations depending on the pH, temperature, and composition of the medium [23]. Our recent work demonstrated that dispersing the freeze-dried mixture of zein with phosphatidylcholine (PC) in aqueous media produces zein-PC nanoparticles (Z/PC-NP) in which the zein core is surrounded by a zein-PC hybrid shell [24]. Zein was present in a positively charged form in Z/PC-NP, and the Z/PC-NP exhibited a higher drug encapsulation capacity than the PC-NP. The serum stability and the storage stability of the Z/PC-NP was greater than that of the PC-NP, and zein functioned as a cryoprotectant when the Z/PC-NP dispersions were freeze-dried [24]. Based on these findings, we herein sought to engineer liposomal ICG by hybridizing PC with zein molecules and investigated the hybridization effect on the ICG stability.

## 2. Materials and Methods

### 2.1. Materials

1,2-dimyristoyl-*sn*-glycero-3-phosphocholine (DMPC) was purchased from Avanti Polar Lipid Inc. (Alabaster, AL, USA). The cholesterol (CHOL) and soybean oil were bought from Sigma-Aldrich (St. Louis, MO, USA). The zein (Zein DP, Showa Sangyo, Tokyo, Japan) was kindly provided by Richwood Trading Company Ltd. (Seoul, Korea). The ICG was obtained from Dongindang Pharmaceutical (Siheung, Kyunggi-Do, Korea). The d-glucose (dextrose) was bought from Amresco (Solon, OH, USA). The 3-(4,5-dimethylthiazol-2-yl)-2,5 -diphenyltetrazolium bromide (MTT) was obtained from Amresco (Solon, OH, USA). All other materials were of reagent grade and used without further purification.

### 2.2. Cell Line and Culture

The human oral cancer A253 cell line was purchased from the Korean Cell Line Bank (Seoul, Korea). The A253 cells were cultured in McCoy’s 5A medium (Gibco, Grand Island, NY, USA) supplemented with 10% heat-inactivated fetal bovine serum (FBS) (Hyclone, Logan, UT, USA) and 100 units/mL each of penicillin and streptomycin (Gibco, Grand Island, NY, USA). The cells were grown in incubators in a humid atmosphere of 95% air and 5% CO_2_ at 37 °C.

### 2.3. Preparation of PC-NP and Z/PC-NP

The PC-NP and Z/PC-NP were prepared by a freeze-drying method as described in our earlier study [24]. Briefly, a total of 14.8~21.8 mg of the lipid mixture (16:3 molar ratio of DMPC and CHOL), 0.85 mg of ICG and 2 µL of soybean oil were dissolved together in approximately 2 mL of tertiary butyl alcohol. Zein (0~7 mg) was separately dissolved in 1 mL of 85% tertiary butyl alcohol. The solutions were mixed, rapidly frozen at −80 °C and subjected to lyophilization by a freeze dryer (FDU-1200, EYELA, Tokyo, Japan) for 24 h. The obtained dried cakes were hydrated with 1 mL of 5% dextrose solution added dropwise with continuous vortex mixing. The hydrated colloidal dispersion was sonicated at 37 °C for 2 h using a bath-type sonicator (3510R-DTH, Bransonic Ultrasonics, Danbury, CT, USA), followed by additional sonication for 7 min by using a cell disruptor (Bioruptor^®^, UCD-200T, CosmoBio, Tokyo, Japan) at a power of 250 W to get an ICG-encapsulated Z/PC-NP dispersion with increased homogeneity. In our preliminary experiment, the prepared Z/PC-NP dispersions were dialyzed against a 5% dextrose solution to remove the free ICG. Since the free ICG was found to be ≤1% of the total ICG, the Z-PC-NP dispersions were used without separating the free ICG in subsequent experiments.

When additional lyophilization of the Z/PC-NP dispersion was required, the Z/PC-NP dispersions were mixed with an equal volume of distilled water containing 10% sucrose, frozen at −80 °C and then subjected to lyophilization overnight. The lyophilized samples were rehydrated with the original volume of distilled water for redispersion.

### 2.4. Physicochemical Characterization of Z/PC-NP

The mean particle size and polydispersity index (PI) of the Z/PC-NP were determined by a dynamic light-scattering method using a fiber-optic particle analyzer (FPAR-1000, Otsuka Electronics, Osaka, Japan). Prior to analysis, the Z/PC-NP dispersions were diluted with filtered deionized water to reach the analytical measurement range, and the obtained dynamic light scattering data were assessed using the CONTIN analysis program provided by the manufacturer. The zeta potential (the electric potential at the shear plane of the nanoparticle) was measured with the diluted Z/PC-NP sample using a Zen 3600 Zetasizer (Malvern Panalytical, Malvern, UK). Default instrument settings and automatic analysis modes were used for all measurements.

The Fourier-transform infrared spectroscopy (FTIR) spectral measurement was performed using a Thermo Scientific Nicolet Nexus 670 FTIR spectrometer and a Smart iTR with a diamond window (Thermo fisher scientific, Waltham, MA, USA). Prior to analysis, the samples were freeze-dried and placed on the spectrometer for collecting the spectra in the 550–4000 cm^−1^ range.

In order to study the size and morphology of the ICG-encapsulated Z/PC-NP, transmission electron microscopy (TEM) images were obtained using negative staining TEM (Tecnai G2 spirit, FEI Company, Hillsboro, OR, USA). The ICG-encapsulated Z/PC-NP samples were placed on 200-mesh copper grids coated with carbon, and stained with 2% aqueous uranyl acetate. After 1 min, excess stain was removed and the specimen was air-dried completely. The images were obtained by the TEM instrument operating at 120 keV.

### 2.5. ICG Stability

To evaluate the formation of the ICG aggregates in an aqueous media, the ICG samples (free ICG solution or ICG encapsulated in Z/PC-NPs with varying Z/PC ratios) corresponding to 0.85 mg/mL of ICG were kept at room temperature under light exposure. At pre-determined time intervals, the absorbance spectrum of each sample was obtained in the 550~950 nm range using a spectrophotometer (DU730, Beckman Coulter, FL, USA).

To evaluate the chemical degradation of ICG, the time-dependent changes in absorbance and fluorescence of the ICG samples (free ICG solution or ICG encapsulated in Z/PC-NPs with varying Z/PC ratios) were monitored. Samples were diluted with 5% dextrose to obtain 0.1 mg/mL of ICG and kept at room temperature under light exposure. At pre-determined time points (0, 1, 2, 4, and 6 days), each sample was diluted 20-fold with ethanol in order to disrupt the nanoparticles. The absorbance was measured at 780 nm using a spectrophotometer, and the fluorescence intensity (FI) was determined at an excitation wavelength of 775 nm and emission wavelength of 805 nm using a fluorescence spectrometer (FS-2, SCINCO, Seoul, Korea).

### 2.6. Photocytotoxicity of ICG

The A253 cells were seeded into 24-well tissue culture plates at a density of 5 × 10^4^ cells per well. Twenty-four hours later, the cells were treated with varying ICG formulations corresponding to 40 µM of ICG. After overnight incubation, the cell media was replaced with phosphate-buffered saline (PBS) to ensure maximum laser penetration. The cells were irradiated with the NIR laser (JM Labtech, Seoul, Korea) at a wavelength of 785 nm for 3 min with a 100 mW/cm^2^ output. After irradiation, the PBS was replaced with a fresh cell culture medium supplemented with 10% FBS. The cells were allowed to grow in a CO_2_ incubator at 37 °C for 48 h. After incubation, the growth and viability of the cells were determined by using MTT. The absorbance was measured using a microplate reader (Bio-TEK, Winooski, VT, USA) at 570 nm after dissolving the formazan crystals formed in the cells in dimethyl sulfoxide.

### 2.7. Statistical Analysis

A two-tailed unpaired student t-test was employed to determine the statistically significant differences between values obtained under different experimental conditions.

## 3. Results

### 3.1. Characterization of ICG-Encapsulated Z/PC-NP

We first checked the effect of ICG encapsulation on the colloidal stability of Z/PC-NP. The mean particle size and the PI shown in Table 1 indicate that mixing the zein with a DMPC:CHOL mixture produced nanosized and homogeneous particles. Encapsulating the ICG in Z/PC-NP did not cause any significant changes in the mean size and the PI of the Z/PC-NP, suggesting that the homogeneity of the Z/PC-NP was not hampered by the ICG encapsulation. The surface charge of the Z/PC-NP changed from a slightly negative value to a highly negative one by the ICG encapsulation. This implies that a significant portion of the ICG, a negatively charged molecule, was incorporated into the membrane of the Z/PC-NP, and the ICG incorporation may provide the electrostatic stabilization of the Z/PC-NP. Moreover, the sizes of the Z/PC-NP did not increase significantly regardless of the ICG encapsulation after lyophilization, indicating that the zein functions as an effective cryoprotectant of zein-phosphatidylcholine (Z/PC), regardless of the ICG encapsulation (Table 1).

**Figure 1:**
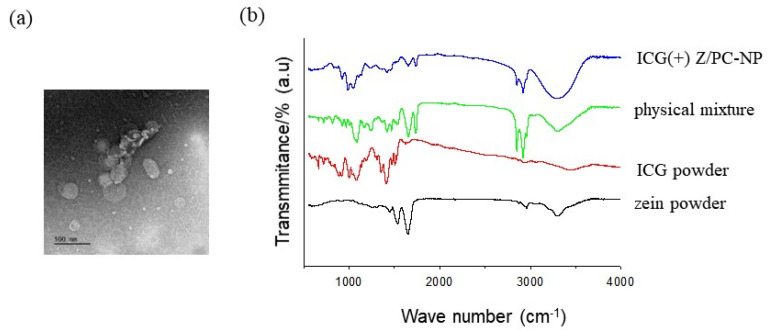
(**a**) A representative negative staining TEM image of ICG-encapsulated Z/PC-NP. Scale bar: 500 nm. (**b**) A FTIR spectrum of zein powder, ICG powder, physical mixture and ICG-encapsulated Z/PC-NP (ICG(+) Z/PC-NP). The Z/PC-NP was prepared from 14.8 mg of lipid (DMPC:CHOL mixture) and 7 mg of zein. The Z/PC-NP samples were freeze-dried before FTIR measurement.

The TEM images of the ICG-encapsulated Z/PC-NP revealed that the particles were spherical with dimensions of 200~300 nm, with these values being similar to those obtained by dynamic light scattering analysis (Figure 1a).

A FTIR spectral measurement was carried out to study the structure of ICG-encapsulated Z/PC-NP further (Figure 1b). With the zein powder, the main bands of the spectrum were obtained at 1539, 1652, and ~3300 cm^−1^, which originate from C=O stretching from amide I, C-N stretching from amide II, and O-H& N-H stretching from amide A, as shown in earlier studies, including our own [24,25]. The spectrum of ICG powder showed several bands related to the aromatic planes of ICG molecules: C-H out-of-plane bending (600–1000 cm^−1^), C-H vinyl stretches (900–1100 cm^−1^), and aromatic C=C stretches (1400–1500 cm^−1^) [5,15]. The physical mixture (ICG, zein, DMPC, CHOL, and SBO) showed typical peaks of PC (the band at 1727 and 2851/2920 cm^− 1^ corresponding to the stretching of the ester C=O and the stretching of the CH_2_ groups of the alkyl chains of PC) [24], in addition to the characteristic peaks of ICG or zein, as described above. Compared to the physical mixture, the peaks corresponding to the aromatic C=C stretches (1400–1500 cm^−1^) and the C-H out-of-plane bending (600–850 cm^−1^) of ICG molecules greatly decreased in the ICG-encapsulated Z/PC-NP. The bands at 1539 and 1653 cm^−1^ corresponding to the stretching of amide bonds of zein molecules and the bands at 1727 and 2851/2920 cm^− 1^ corresponding to PC also decreased in the Z/PC-NP. These results indicate that the motion of ICG, PC, and zein molecules was restricted by entrapment in nanoparticles, confirming the formation of ICG-encapsulated Z/PC-NP. In addition, the band at ~3350 cm^− 1^, attributable to the O-H stretching of water molecules hydrogen-bonded to PC molecules [24,26], appeared broadened with a higher intensity in the Z/PC-NP spectrum compared to that of the physical mixture. This implies that the heterogeneous hydrogen bond interactions increased because of the co-insertion of ICG and zein in the shell of the Z-PC-NP.

**Figure 2:**
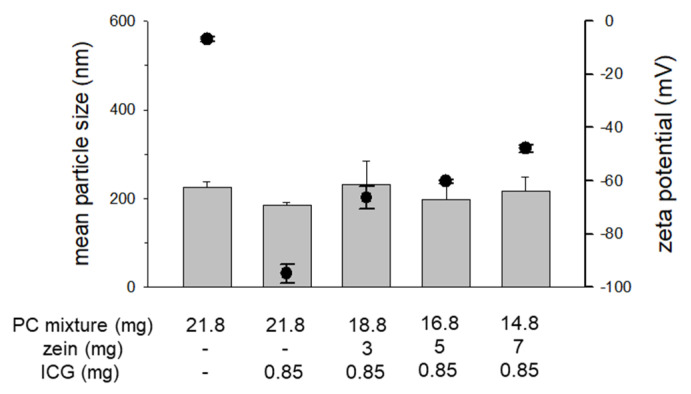
The effect of the zein-to-PC ratio on the mean particle size and the zeta potential of Z/PC-NP. The closed circle symbols indicate the zeta potential and the bars indicate the mean particle size.

To investigate the effect of the zein to PC ratio on the colloidal stability of ICG-encapsulated Z/PC-NP, we prepared the NP with varying zein-to-PC ratios. Figure 2 shows that the mean particle sizes of the Z/PC-NP were approximately 200 nm regardless of the zein-to-PC ratio. The surface charge of the ICG-encapsulated Z/PC-NP showed a tendency toward increased negativity with increases in the zein content (decrease in the PC content). These data suggest that the portion of ICG exposed on the surface of the NP was decreased by the zein-PC hybridization. When considering that the Z/PC-NP possess an inner zein core as presented in our earlier study [24], it seems likely that the accommodation of ICG inside of the NP increased through increased ICG-zein interaction in the Z/PC-NP with a higher zein content.

### 3.2. Effect of Z/PC-NP Encapsulation on ICG Stability

**Figure 3:**
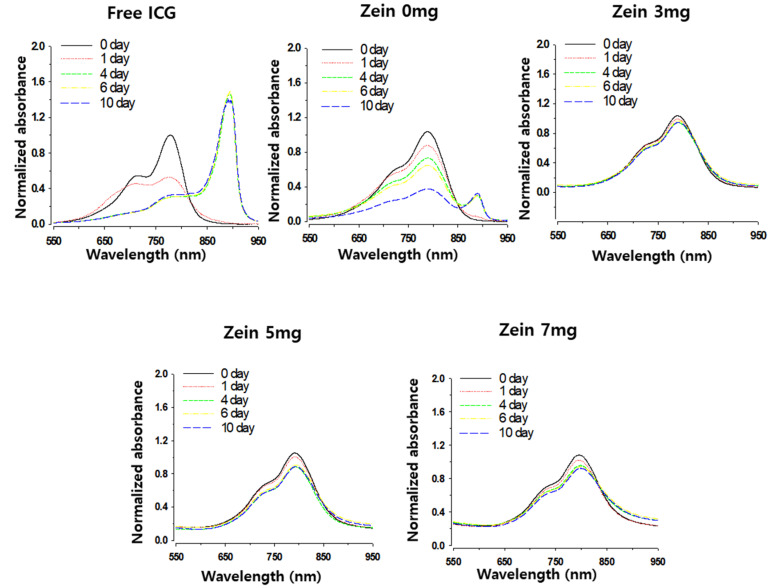
The effect of zein hybridization on the time-dependent formation of ICG J-aggregates. Changes in the absorbance spectrum of varying ICG formulations (0.85 mg/mL ICG concentration) were determined during incubation at room temperature. Free ICG solution was prepared in 5% D-glucose. The spectrum was obtained after sample dilution with 5% D-glucose and normalized by the 780 nm absorbance of each sample on the initial day.

We first checked the effect of zein-PC hybridization on ICG stability in terms of aggregate formation. ICG is known to readily form highly ordered aggregates (J-aggregates) in an aqueous medium, causing variability in the maximum absorbance wavelength and a great decrease in emission intensity [8,27]. Compared to the absorbance spectra of the free ICG solution, the spectra of the ICG encapsulated in Z/PC-NP were red-shifted (9, 10, 11, and 17 nm shift for zein 0, 3, 5, and 7 mg nanoparticles, respectively) (Figure 3). These data indicate ICG entrapment and an affinity of ICG for zein within the nanoparticles (NP). During the 10-day storage of the free ICG solution, its absorption peak at 780 nm decreased in a time-dependent manner. Concurrently, a new absorption peak at 894 nm appeared and increased, which indicates the formation of ICG J-aggregates [8]. ICG encapsulated in PC-NP (zein 0 mg) tended to form a smaller but still evident peak at 894 nm at 10 days post-incubation, indicating the partial inhibition of aggregate formation. In contrast, the ICG encapsulated in Z/PC-NP (zein 3~7 mg) did not show A894 peaks during the 10-day incubation. This indicates that the Z/PC-NP inhibited the aggregation of the ICG much more effectively than PC-NP. Presumably, the ICG–zein interaction in the Z/PC-NP decreased the interactions between the ICG molecules, leading to aggregation.

**Figure 4:**
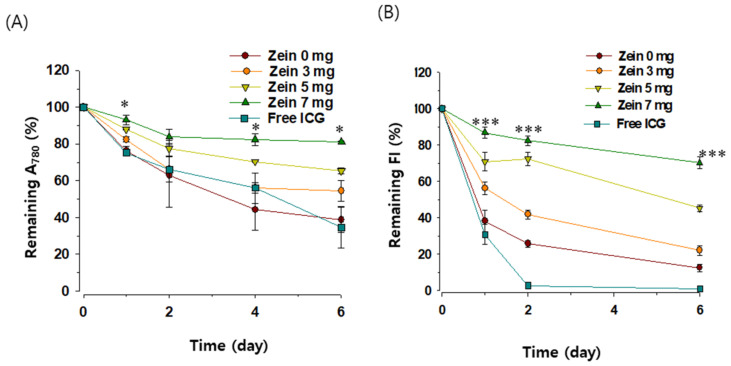
The effect of zein hybridization in PC-NP on the time-dependent degradation of encapsulated ICG. ICG degradation was assessed by changes in absorbance (**A**) and FI (**B**) of ICG (0.1 mg/mL) during storage at room temperature. The value on day 0 was set at 100%. Each point represents the mean ± SD; *n* = 3. Significant differences between the PC-NP with or without zein hybridization are indicated by asterisks: * *p* < 0.05; *** *p* < 0.001.

The saturation of double bonds in the conjugated chain of ICG is known to induce ICG degradation in an aqueous solution, an effect that is accelerated at lower ICG concentrations [28]. The impact of zein-PC hybridization on ICG degradation was assessed using diluted samples (0.1 mg/mL ICG). As shown in Figure 4a, the A_780_ of the free ICG solution rapidly decreased during the six day incubation, losing more than 65% of the A_780_ of the initial ICG after the six day incubation. A_780_ of the ICG encapsulated in PC-NP decreased to an extent similar to the free ICG (61% decrease after the six-day incubation). In contrast, A_780_ of the ICG encapsulated in Z/PC-NP decreased slower with the zein-to-PC ratio increase: the A_780_ of ICG in the Z/PC-NP containing 3, 5, and 7 mg of zein remaining after the six day incubation was 55, 65, and 81%, respective of the initial value (Figure 4a). The FI decrease was the fastest in the free ICG solution: at two days post-incubation, <2% of FI of the initial ICG remained (Figure 4b). The rate of FI decrease in the ICG encapsulated in Z/PC-NP decreased with increases in the zein-to-PC ratio. The FI of the ICG encapsulated in Z/PC-NP containing 0, 3, 5, and 7 mg of zein remaining after two day incubation was 26, 42, 72, and 85%, respectively, of the initial value. In the case of the Z/PC-NP containing 7 mg of zein, 70% of the FI of the ICG still remained six days post-incubation. These results demonstrate that the ICG degradation was only partially protected in the PC-NP and the effectiveness of the protection was increased further by the zein hybridization in the PC-NP.

### 3.3. Photocytotoxicity of ICG

**Figure 5:**
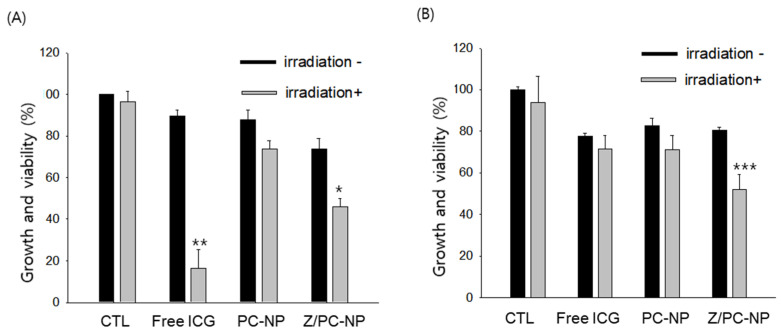
The effect of zein hybridization in PC-NP on the photocytotoxicity of encapsulated ICG. Each ICG formulation was used for treatment on the day of preparation (**A**) or after a four-day storage at room temperature (**B**). PC-NP and Z/PC-NP were prepared from 21.8 mg of lipid (DMPC:CHOL mixture) and 14.8 mg of lipid and 7 mg of zein mixture, respectively. Significant differences between treatments without irradiation or with irradiation are indicated by asterisks: * *p* < 0.05; ** *p* < 0.01; *** *p* < 0. 001.

The effect of zein hybridization in PC-NP on the photocytotoxic activity of encapsulated ICG was investigated in human oral cancer A253 cells, since photodynamic therapy has been regarded as a promising therapeutic strategy, particularly for disorders in the oral cavity [29]. The A253 cells were treated with each ICG formulation (40 μM ICG) for 16 h, with or without subsequent NIR laser irradiation for 3 min. The ICG concentration and the incubation time were selected based on our recent works [7,19]. When the viability of the cells was determined at 48 h post-incubation, NIR irradiation itself did not affect the cell viability. Upon NIR irradiation, the free, fresh ICG solution decreased the viability of the A253 cells most greatly, probably due to the rapid uptake of amphiphilic ICG into the cells (Figure 5A). ICG encapsulated in Z/PC-NP also decreased the cell viability significantly upon irradiation, whereas that in PC-NP did not. In this regard, the cellular uptake of ICG encapsulated in PC-NP was found to be very low in our earlier study [19]. When considering the difference in the zeta potential values of PC-NP and Z/PC-NP formulations (−97.9 mv vs. −47.9 mV), the higher photocytotoxicity of the Z/PC-NP formulation compared to the PC-NP formulation may be attributable to the higher cellular uptake of ICG in the Z/PC-NP by reduced electrostatic repulsion with negatively charged cell membranes.

To investigate whether the Z/PC-NP formulation retained the photocytotoxicity of encapsulated ICG longer due to the superior stabilizing efficacy of Z/PC-NP, the photocytotoxicity of ICG was determined after a four-day storage of each ICG formulation. Neither the free ICG nor the PC-NP formulation inhibited the growth of A253 cells significantly upon irradiation. In contrast, the Z/PC-NP formulation inhibited the cell proliferation significantly (Figure 5B), which can be attributable to the retained photosensitizing activity of ICG encapsulated in Z/PC-NP.

## 4. Conclusions

ICG-encapsulated zein-PC hybrid nanoparticles exhibited high colloidal stability. More importantly, zein hybridization in PC-NP greatly improved the ICG stabilization achieved by the PC-NP encapsulation. The Z/PC-NP encapsulation enabled the photocytotoxicity of the encapsulated ICG retained after storage. Taken together, our findings highlight the promising potential of zein-PC hybrid nanoparticles as an ICG formulation for the phototherapy of cancer.

## Figures and Tables

**Table 1:** The effect of indocyanine green (ICG) encapsulation on the mean particle size and polydispersity index (PI) of zein-phosphatidylcholine hybrid nanoparticles (Z/PC-NP). The Z/PC-NP was prepared with 14.8 mg lipid (1,2-dimyristoyl-*sn*-glycero-3-phosphocholine (DMPC): cholesterol (CHOL) mixture) and 7 mg zein. The prepared Z/PC-NP dispersion was subjected to lyophilization and redispersion as described in the methods section. The data are presented as the mean ± SD (*n* = 3).

ICG	Zeta Potential (mV)	Without Lyophilization		After Lyophilization	
		Mean Size (nm)	POLYDISPERSITY Index	Mean Size (nm)	Polydispersity Index
No	−9.3 ± 1.5	229 ± 36	0.216 ±0.047	281 ± 48	0.311 ± 0.035
Yes	−47.9 ± 1.4	216 ± 33	0.210 ± 0.029	247 ± 74	0.219 ± 0.036

## Data Availability

Data is contained within the article.
